# Book review

**DOI:** 10.3402/ejpt.v6.27793

**Published:** 2015-04-28

**Authors:** Joanna Oughton

**Affiliations:** University of Nottingham, England


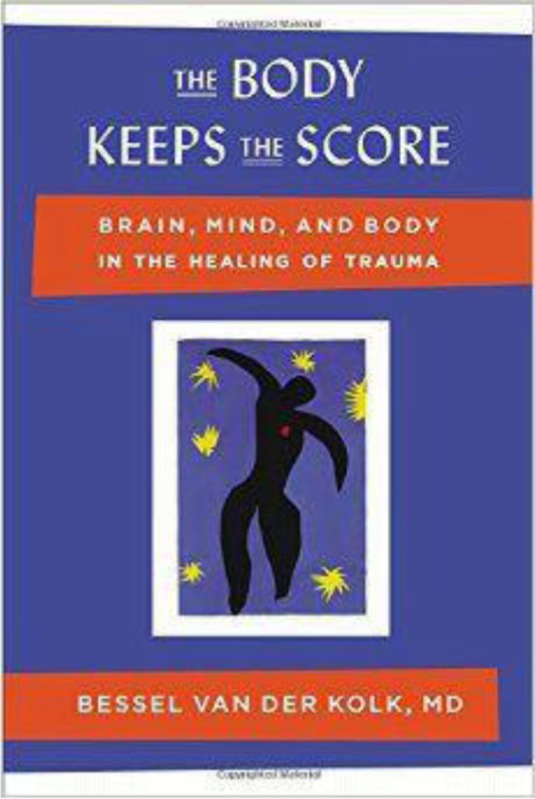


Review of *The body keeps the score*, by Bessel van der Kolk. New York: The Penguin Group, 2014. 443 pp. ISBN: 978-670-78593-3. $27.95 Hardback.

An astonishing amount of information on almost every aspect of trauma experience, research, interventions, and theories is brought together in this book, which, despite the author's medical background as a psychiatrist, has a distinctly holistic feel to it. The title suggests that what will be explored is how the body retains the imprints of trauma. However, it delivers much more than this, delving into how the brain is impacted by overwhelming traumatic events, and is studded with sections on neuroscience which draw on the author's own numerous studies as well as that of his peers. In addition, it investigates the effects of adverse childhood attachment patterns, child abuse, and chronic and long-term abuse.

The book is divided into five sections, each one building historically from war fever, shell shock, and early posttraumatic stress disorder diagnosis to up-to-date research, trauma interventions, and possible pathways to recovery. An entire section examines the effects of abnormal attachment in children and adults and also discusses child abuse, as well as gives an impassioned argument for the creation of and inclusion to the DSM diagnostics of a new diagnosis of *developmental trauma disorder* to enable clinicians to better diagnose the cumulative effects of chronic child abuse, and also to focus and enable research to be undertaken in this area. Van der Kolk cites his own and other research studies that show clear differences between the effects of traumatization as an adult and as a child, and an appendix outlines the proposed criteria.

This book is written in a very accessible style. In the first chapter, the author focuses on the increased understanding of trauma that came from the returning Vietnam Veterans who brought with them a new form of combat trauma, resulting in the subsequent diagnostic category of posttraumatic stress disorder. Each chapter is written eloquently and has case studies involving the author's patients, bringing each topic very much to life.

The section on the brain and body was punctuated with drawings and diagrams, but again it was case studies such as that of the couple caught in a horrific multiple car pileup and the subsequent research by Dr Ruth Lanius using fMRI scans to track neural activity of flashbacks, which gave insight into the impact of trauma on brain function, that really helped to reinforce the points that the author was trying to make.

Many times, the author's humanity is apparent, for example, as he argues against the standard practice of attributing to traumatized children multiple labels such as “oppositional defiant” and describing them as vengeful, impulsive, and risk taking by saying *we can only help if we correctly define what is going on with them*, that they are scared to death in a body that is constantly being tricked by a faulty alarm system into believing that the whole world is against them. There was so much information covered that it could potentially have been better fitted in a series of books, giving the author the opportunity to share in more detail his obvious wealth of knowledge about each of the subject areas.

The section on children is followed by an exploration of the nature and cause of traumatic memory, and Van der Kolk then devotes the final part of the book to the many possible paths to recovery, with chapters on yoga, Eye Movement Desensitization Reprocessing (EMDR), and neurofeedback, among others.

It is in the final section of the book that the author suggests that there is no way to treat trauma, and that only the imprint, the flashbacks, the nightmares, loss of control, overwhelm and shutdown, and so on, can be treated by finding a way to stay calm, focused, and respond to the images and sensations that arise. He states the need to restore equilibrium in the limbic system and argues that self-awareness and emotional regulation are vital to recovery. He highlights breath work to engage the parasympathetic nervous system, neurofeedback for shutdown and hyperarousal, mindfulness to bring body awareness and an observing quality to the traumatic experience and emotions, as well as a vital need for a good support network and a sense of safety.

He talks at various times of his lack of belief in a number of well-utilized interventions, such as cognitive behavioral therapy (CBT) and exposure therapy, which primarily work on avoidance, arguing that critical areas of the brain are knocked out during reactivation of the trauma which cause overwhelm, making it difficult, if not impossible, to integrate the traumatic material.

Indeed, the author continues these arguments in the chapter “Language in the Paths to Recovery,” stating that psychoanalytic therapy and CBT were the only two approaches recommended by an expert panel in the aftermath of the twin towers as Manhattan has strong connections to psychoanalysis and CBT as it can be manualized and is the popular treatment with academic researchers. He highlights the lack of research into lesser known or not so popular therapies while continuing to build arguments for them, stating that trauma survivors actually list massage, yoga, and EMDR as the most beneficial in dealing with their traumatic experiences.

While many may agree that there needs to be greater research into these other interventions, there may also be many who disagree with the author's conclusions about talk therapies and CBT, as there is a lot of documented evidence in support of CBT, particularly trauma-focused CBT. Whichever side trauma professionals come down on, his discussions just seemed to open up further areas of dialog and potential for research, all of which can ultimately only benefit the people whose lives are impacted by traumatic events.

Overall, the book gives the impression that the author is a trauma specialist who genuinely cares for the people he treats, and whose strong desire to alleviate their suffering has resulted in a lifetime's work to try and find answers and solutions to their many problems, resulting in this book, which would give the reader, both professional and layperson, a sound overview of most of the prominent areas of trauma research. Traumatized individuals may find some of the case study descriptions a little challenging, but for anyone interested in discovering the umbrella of trauma work, this book is a veritable goldmine of information.

*Joanna Oughton* University of Nottingham, England

